# Empirically Derived Dietary Patterns and Health-Related Quality of Life in the SUN Project

**DOI:** 10.1371/journal.pone.0061490

**Published:** 2013-05-01

**Authors:** Cristina Ruano, Patricia Henriquez, Miguel Ángel Martínez-González, Maira Bes-Rastrollo, Miguel Ruiz-Canela, Almudena Sánchez-Villegas

**Affiliations:** 1 Department of Clinical Sciences, University of Las Palmas de Gran Canaria, Las Palmas, Spain; 2 Department of Preventive Medicine and Public Health, University of Navarra, Pamplona, Spain; 3 Ciber Fisiopatología Obesidad y Nutrición (CIBEROBN, CB06/03), Instituto de Salud Carlos III, Madrid, Spain; The Ohio State University, United States of America

## Abstract

**Objective:**

The analysis of dietary patterns has become a valuable tool to examine diet-disease relationships but little is known about their effects on quality of life. Our aim was to ascertain the association between major dietary patterns and mental and physical quality of life after 4 years of follow-up.

**Materials and Methods:**

This analysis included 11,128 participants from the “Seguimiento Universidad de Navarra” (SUN) cohort. Dietary habits were assessed using a validated food-frequency questionnaire. Factor analysis was used to derive dietary patterns. Quality of life was measured with the validated Spanish version of the SF-36 Health Survey.

**Results:**

Two major dietary patterns were identified, the ‘Western’ dietary pattern (rich in red meats, processed pastries and fast-food) and the “Mediterranean” dietary pattern (high in fruits, vegetables and olive oil). After controlling for confounders, the Western dietary pattern was associated with quality of life in all domains. The magnitude of these differences between the subjects in the highest (quintile 5) and the lowest quintile of adherence to the Western pattern ranged from −0.8 (for mental health) to −3.5 (for vitality). On the contrary, the Mediterranean dietary pattern was associated with better quality of life domains: differences ranged from +1.3 (for physical functioning) to +3.4 (for vitality) when comparing extreme quintiles of adherence. Additional sensitivity analyses did not change the reported differences.

**Conclusions:**

Whereas baseline adherence to a Western dietary pattern was inversely associated with self-perceived quality of life after 4 years of follow-up, baseline adherence to a Mediterranean dietary pattern was directly associated with better scores in quality of life four years later in the SUN Project.

## Introduction

Population ageing has fostered the general concern for obtaining a better health-related quality of life (HRQL). HRQL is a multidimensional concept that refers to the physical, psychological and social domains of health [1]. Each of these domains has different components to be measured and they can represent both an objective (functioning and health status) and subjective (perceptions) dimension of health. The measuring of HRQL has been frequently applied to patients but it is also interesting to assess it among healthy subjects. Several factors are well-known determinants of HRQL [2]–[4], some of them are related to lifestyle, and therefore they are modifiable. Among them, dietary habits are especially interesting. Beyond isolated food items, the assessment of overall dietary patterns is likely to provide a better explanation of diet-health associations.

The application of dietary patterns has become of considerable interest in nutritional epidemiology [5], [6]. The idea that persons do not consume isolated foods or nutrients but include them in a varied overall dietary pattern and that food and nutrients can have synergistic or antagonistic effects when they are consumed together has had a growing acceptance in nutritional epidemiology during the last decade. The effect of an isolated nutrient could be too small as to be able to be detected, whereas the cumulative effect of multiple nutrients included in an overall dietary pattern can be sufficiently large as to exhibit sizable health effects. On the other hand, the close correlation between some nutrients could complicate to study them as separate entities. In addition, if the intake of several nutrients is associated with some dietary patterns, the analysis of an isolated nutrient could be biased by the effect of the overall dietary pattern.

The scientific literature has consistently shown the effects of certain diets on health. The deleterious effects of a “Western-type” [7]–[9] and the benefits of a traditional Mediterranean diet have been consistently described in many epidemiological studies [10]–[12]. However, few studies have analyzed the influence of diet on the quality of life of healthy populations [13], [14]. In a recent report from our cohort we have shown that the adherence to a Mediterranean Diet assessed by an a priori approach (Hypothesis-oriented food pattern) was associated with better scores in self-perceived quality of life [15]. Our results were in accordance with another study also conducted in Spain which showed the same beneficial effects of the adherence to this dietary pattern on quality of life [16].

However, an alternative approach to ascertain dietary patterns is the use of principal component analysis to obtain empirically-derived patterns (a posteriori approach). This alternative has been shown to be a powerful method for summarizing nutrient and food intake to depict the whole diet and it also has the advantage of reflecting existing food habits in the study population. Moreover, the results can be easily translated into public health recommendations [5], [17].

Thus, the purpose of the present study was to assess the association between baseline adherence to empirically-derived dietary patterns and self perceived physical and mental HRQL collected after 4-year follow up in the SUN cohort.

## Materials and Methods

### Study population

The “Seguimiento Universidad de Navarra’’ (SUN) Project is an ongoing, multipurpose, dynamic cohort of university graduates conducted in Spain and started in December 1999. The study methods and the cohort profile have been published in detail elsewhere [18], [19].

**Figure 1:**
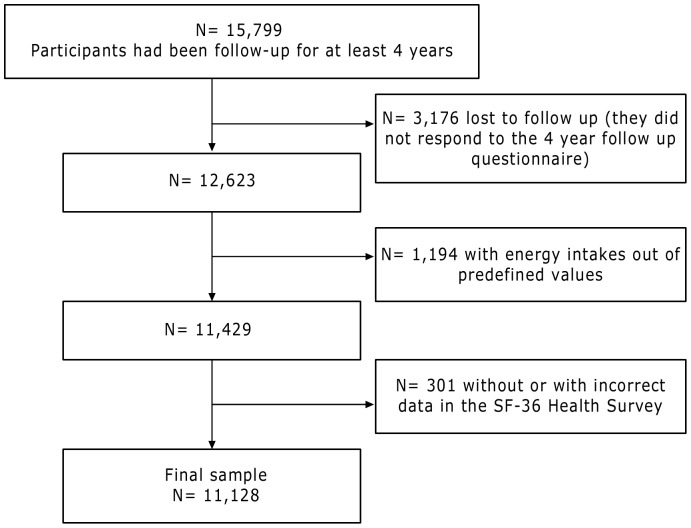
Flowchart of participants in the present analysis.

For this analysis, we included participants who had already been followed-up for at least 4 years (n  = 15,799). Among them, 12,623 were successfully followed-up for at least 4 years. We excluded those participants who were outside of predefined limits for total energy intake (<800 kcal/d in men and <500 kcal/d in women or >4000 kcal/d in men and >3500 kcal/d in women) (n = 1,194) [20], and participants without or with incorrect data regarding quality of life (n = 301). After exclusions, 11,128 participants remained available for the analyses (Figure 1).

The study was approved by the Human Research Ethical Committee at the University of Navarra. Voluntary completion of the first questionnaire was considered to imply informed consent and our Committee specifically approved this consent process.

### Exposure assessment

Dietary intake was assessed using a semi-quantitative food frequency questionnaire (136 food items) completed at baseline [21]. Validity and reproducibility of this questionnaire has recently been re-evaluated [22], [23]. For example, it showed reasonably good validity for assessing the different food groups (intraclass correlation coefficients versus four 3-day food records ranged from 0.40 to 0.84).

A trained dietician updated the nutrient data bank using the latest available information from the food composition table for Spain [24], [25]. The 136 food items included in the semi-quantitative food frequency questionnaire were classified in 20 predefined food groups (Table 1). The grouping scheme was based on the similarity of nutrients profile or culinary usage among the foods and was somewhat similar to that used in other studies [26]. A principal component analysis was applied to these groups in order to identify a reduced number of factors that could explain the maximum proportion of the variance from the original groups [27]. The factors were rotated by Varimax orthogonal transformation to achieve a simpler structure with greater interpretability. In determining the numbers of factor to retain, we considered the Scree test [28], and the interpretability of the factors.

**Table 1:** Food groupings used in the dietary pattern.

Food or food groups	Food items
Vegetables	Carrots, swiss chard, cauliflower, lettuce, tomatoes, green beans, eggplant, peppers, asparagus, spinach, other fresh vegetables
Fruits	Citrus, banana, pear, strawberry, peach, cherry, fig, melon, watermelon, grapes, kiwi, mango
Legumes	Lentils, chickpeas, beans, peas.
Refined cereals	White bread, cold breakfast cereals, rice, pasta.
Whole-wheat bread	Whole-wheat bread
High-fat dairy products	Whole milk, condensed milk, cream, milk shake, yogurt, custard, cheese, crème caramel, ice-cream, other dairy products
Low-fat dairy products	Skim or low-fat milk, skim yogurt, white cheese
Poultry	Chicken, turkey, rabbit.
Red and processed meats	Beef, pork, lamb, liver, cooked ham, Parma ham, mortadella, salami, foie-gras, spicy pork sausage, bacon, cured meats and cold cuts
Nuts	Walnuts, peanuts, almonds, hazelnuts, date, raisins
Eggs	Eggs
Fish and other seafood	White fish, dark-meat fish, salad or smoked fish, clams, mussels, shrimp, squid
Animal fats	Butter, lard
Vegetable oils and fats	Margarine, sunflower oil, corn oil
Commercial bakery goods	Muffins, doughnuts, croissants, other industrial bakery
Processed food	Croquettes, ready-made soup and other processed foods
Fast food	Hamburger, pizza, hot-dog, French fries
Olive oil	Olive oil
Sauces	Mayonnaise, tomato sauce, ketchup
Potatoes	Cooked or roast

Food groups with absolute loading >0.30 were considered relevant components of the dietary patterns (Table 2). Food groups such as refined cereals, whole-wheat bread, nuts, animal fats and vegetable oils and fats, with absolute loadings lower than 0.30, were excluded from the final model.

**Table 2:** Factor loading matrix for the major factors (diet patterns) identified by using food consumption data from the FFQ used in the SUN cohort.

	Factor 1	Factor 2
Food or food group	(Western Dietary Pattern)	(Mediterranean Dietary Pattern)
Vegetables		0.695
Fish and other seafood		0.566
Fruits		0.552
Poultry		0.359
Olive oil		0.358
Potatoes		0.358
Low-fat dairy products		0.357
Legumes		0.307
Fast food	0.644	
Red and processed meats	0.581	
High-fat dairy products	0.479	
Processed food	0.444	
Eggs	0.409	
Commercial bakery goods	0.387	
Sauces	0.383	

Absolute values <0.30 were not included in the table for simplicity.

We used the factor loading matrix to extract the weights (factor loadings) for each food group. Each pattern was constructed by summing standardized intakes of the component food items weighted by factor scores [28].

### Outcome assessment

Quality of life was assessed after the 4-year follow-up with the validated Spanish version of the SF-36 Health Survey. The SF-36 is a general health scale widely used and thoroughly validated [29]. This instrument has been translated into a number of languages, including Spanish. The Spanish weights obtained were very similar to those of the original American version (correlation coefficients >0.9) [30]. The questionnaire contains 36 items which measure eight multi-item domains of health status: 1) physical functioning, 2) role limitations due to physical health problems (role-physical), 3) bodily pain, 4) general health perceptions, 5) vitality, 6) social functioning, 7) role limitations due to emotional problems (role emotional) and 8) mental health. Domains 1 to 4 of the questionnaire deal with physical aspects, while domains 5 to 8 measure psychological features. For each parameter, scores were coded, summed and transformed to a scale from 0 (the worst possible condition) to 100 (the best possible condition). In the case of the bodily pain domain a score of 100 means a complete tolerance or absence of pain.

The raw domain scores were standardized and aggregated into two summary measures: Mental Summary Component, summarizing the 4 mental domains, and Physical Summary Component, summarizing the 4 physical domains [30].

### Covariate assessment

The baseline assessment also gathered information on socio-demographic variables, anthropometric variables, lifestyle and health-related habits, and medical history [19]. Self-reported anthropometric variables were previously validated in a subsample of the cohort [31].

At baseline, participants also completed a validated physical activity questionnaire that collects information about 17 activities [32]. Leisure-time activities were computed by assigning an activity metabolic equivalent (MET) score to each activity, multiplied by the time spent in each activity and summing up all activities [33].

### Statistics

Generalized Linear Models were used to assess the relationship between quintiles of adherence to both dietary patterns and the different domains of the SF- 36. We estimated multivariable-adjusted means and their 95% confidence intervals (95% CI) for each quintile. To ascertain if these means were significantly different by pairs, a post hoc correction for multiple testing was used (Benjamini-Hochberg procedure).

We also estimated the regression coefficients (95% CI) of the mental and physical summary components for the four upper quintiles using the lowest quintile as the reference category. Potential confounders included as covariates in the models were: age (years, continuous), sex, smoking (never, past and current smokers), physical activity during leisure time (METS-h/week, quintiles), total energy intake (Kcal/day, continuous), BMI (kg/m^2^, continuous), and prevalence of cardiovascular disease (CVD), diabetes, dyslipidaemia and hypertension at baseline. Additionally, tests of linear trend across successive quintiles of adherence were conducted assigning the median value to each quintile category and treating the variable as continuous.

Differences in the eight parameters of the SF-36 according to the level of adherence to the dietary patterns can be assessed along two concepts: clinical and statistical differences. Clinically significant differences are defined as a 5-point difference in the 0–100 scale [34], whereas statistically significant differences were defined as 2-tailed p<0.05.

Finally, several sensitivity analyses were carried out to assess possible sources of bias in the estimation of the association between adherence to the WPS or the MPS and the SF-36 health domains. We repeated the analyses after: 1) adopting different allowed limits for total energy intake, 2) excluding participants with CVD, diabetes and cancer at baseline, 3) additionally adjusting for socio-economic variables: years of education, marital status and employment status, 4) adjusting for alcohol intake, 5) repeating the factor analysis including 25 predefined food groups or 30 predefined food groups.

The SPSS software package for Windows version 19.0 (SPSS Inc., Chicago, IL) was used for statistical analyses.

## Results

Factor analysis revealed two major dietary patterns accounting for 14.1% of the total variance. The first dietary pattern could typify a Western dietary pattern (WDP). This pattern was characterized by high consumption of fast food, red and processed meats, high-fat dairy products, processed foods, refined cereals, eggs, commercial bakery goods, and sauces. The second dietary pattern was labelled as “Mediterranean dietary pattern” (MDP) and was characterized by high consumption of vegetables, fish and other seafood, fruits, poultry, olive oil, potatoes, low-fat dairy products, and legumes.

Younger subjects, men, current smokers and those with higher total energy intake were more likely to belong to the highest quintile of adherence to the WDP (Table 3). In contrast, subjects in the highest quintile of adherence to MDP were more likely to be women, and more physically active.

**Table 3:** Baseline characteristics of participants according to extreme quintiles of the two major dietary patterns in the SUN project.

		
	Western dietary pattern			Mediterranean dietary pattern		
	Q1	Q5	p value	Q1	Q5	p value
	(n = 2225)	(n = 2225)		(n = 2225)	(n = 2225)	
Sex (% men)	32	60	<0.001*	53	32	<0.001*
Age at baseline (years), mean (SD)	42 (12)	38 (11)	<0.001†	37 (11)	34 (10)	<0.001†
BMI (kg/m^2^), mean (SD)	24 (3)	23 (3)	0.320†	23 (3)	24 (3)	0.812†
Smoking						
Ex smoker (%)	39	23	<0.001*	27	34	<0.001*
Current smoker (%)	19	25	<0.001*	25	20	<0.001*
Leisure time physical activity (Mets-h/week), mean (SD)	22 (23)	21 (23)	<0.001†	17 (19)	24 (26)	<0.001†
Total energy intake (Kcal/d), mean (SD)	1886 (569)	2948 (469)	<0.001†	1982 (616)	2737 (513)	<0.001†
History of diseases (%)						
Diabetes	3	1	<0.001*	1	2	<0.001*
Hypertension	10	5	<0.001*	6	6	<0.001*
Coronary Heart Disease	6	4	<0.001*	3	9	<0.001*
Dyslipidaemia	27	12	<0.001*	16	22	<0.001*

Continuous variables are expressed as the mean and (standard deviation). Categorical variables are expressed as percentages.

* p value from x^2^ test.

† p value from Student t test.

Table 4 shows the estimated multivariate-adjusted means (95% CI) for the 8 domains of the SF-36 according to quintiles of adherence to the WDP. Mean values for the 8 domains were significantly worse in participants with higher adherence to the WDP with a significant inverse dose-response trend for all the domains.

**Table 4:** Mean scores (95% CI)* of the SF-36 dimensions according to quintiles of Western dietary pattern in the SUN project.

	Quintiles of Western Dietary Pattern					
SF-36 scores after 4 years of follow-up	Q1 (lowest)	Q2	Q3	Q4	Q5 (highest)	p linear trend
	(n = 2225)	(n = 2226)	(n = 2226)	(n = 2226)	(n = 2225)	
**Vitality**	65.2 (63.6–66.8)	64.4 (62.8–65.9)	63.4# (61.8–64.9)	62.6^+#^ (61.0–64.2)	61.7‡ (60.1–63.4)	<0.001
**Social functioning**	89.5 (88.1–90.9)	89.4 (97.9–90.8)	89.5 (88.1–90.9)	88.7 (87.3–90.2)	88.0 (86.6–89.5)	0.004
**Role emotional**	85.8 (82.9–88.6)	85.9 (83.1–88.7)	86.5 (83.7–89.3)	85.1 (82.3–87.9)	83.2 (80.2–86.1)	0.01
**Mental Health**	75.9 (74.9–77.3)	75.6 (74.2–77.0)	75.2 (73.8–76.6)	74.7# (73.3–76.1)	75.1 (73.6–76.5)	0.04
**Physical functioning**	90.2 (89.3–91.2)	90.3 (89.3–91.2)	89.7 (88.7–90.6)	89.4+ (88.5–90.3)	89.1+ (88.2–90.1)	<0.001
**Role physical**	86.3 (83.6–88.9)	86.2 (83.6–88.9)	86.8 (84.2–89.4)	85.4 (82.8–88.0)	84.4 (81.7–87.1)	0.04
**Bodily pain**	75.6 (73.5–77.6)	74.6 (72.6–76.6)	75.4 (73.4–77.4)	73.9 (71.8–75.9)	73.3 (71.2–75.4)	0.003
**General Health**	65.8 (64.2–67.4)	65.1 (63.5–66.7)	64.5 (62.9–66.1)	63.8# (62.2–65.4)	63.2# (61.6–64.8)	<0.001

* Adjusted for age, sex, smoking, leisure time physical activity, total energy intake, baseline BMI and history of hypertension, diabetes, dyslipidaemia and CVD.

‡ statistically significantly lower (p<0.05) than lower quintiles (Q1 to Q4) (Benjamini -Hochberg correction).

+ statistically significantly lower (p<0.05) than Q2 (Benjamini -Hochberg correction).

# statistically significantly lower (p<0.05) than Q1 (Benjamini -Hochberg correction).

After applying the Benjamini-Hochberg post-test correction, statistically significant differences were found in vitality score for the highest (Q5) versus the lowest quintile (Q1) of adherence with an adjusted difference of −3.5 points. Also, for general health, mean scores for subjects in Q5 were significantly worse than those found in Q1. The magnitude of the domains differences between the subjects with the highest and with the lowest adherence ranged from −0.8 (for mental health) to −3.5 (for vitality).

Table 5 shows the estimated multivariate-adjusted means (95% CI) for the 8 domains of the SF-36 according to quintiles of adherence to the MDP. Mean values for vitality, mental health, physical functioning, bodily pain and general health domains were significantly better in participants with higher adherence to the MDP with a significant dose-response trend for each of these domains (p for trend<0.01 0.05). Differences ranged from 1.3 (for physical functioning) to 3.4 (for vitality) when comparing extreme quintiles of adherence.

**Table 5:** Mean scores (95% CI)* of the SF-36 dimensions according to quintiles of Mediterranean dietary pattern in the SUN project.

	Quintiles of Mediterranean Dietary Pattern					
SF-36 scores after 4 years of follow-up	Q1 (lowest)	Q2	Q3	Q4	Q5 (highest)	p linear trend
	(n = 2225)	(n = 2226)	(n = 2226)	(n = 2226)	(n = 2225)	
**Vitality**	61.5 (59.8–63.1)	61.9 (60.3–63.5)	63.7# (62.1–65.3)	64.2^+#^ (62.6–65.8)	64.9† ^+#^ (63.3–66.5)	<0.001
**Social functioning**	83.4 (80.6–86.3)	84.4 (81.6–87.2)	85.8 (82.9–88.5)	86.3 (83.5–89.1)	85.6 (82.8–88.4)	0.182
**Role emotional**	88.5 (87.0–89.9)	88.6 (87.2–90.1)	89.1 (87.7–90.5)	89.5 (88.1–90.9)	89.2 (87.8–90.6)	0.090
**Mental Health**	74.1 (72.6–75.6)	74.7 (73.3–76.1)	75.5# (74.1–76.9)	75.8# (74.4–77.2)	75.8# (74.4–77.2)	0.005
**Physical functioning**	88.8 (87.8–89.7)	89.2 (88.3–90.1)	89.9^+#^ (89.0–90.9)	90.2^+#^ (89.2–91.1)	90.1^+#^ (89.2–91.0)	0.001
**Role physical**	83.9 (81.2–86.6)	85.4 (82.7–88.0)	86.5 (83.9–89.1)	86.5 (83.9–89.1)	85.9 (83.4–88.6)	0.197
**Bodily pain**	72.8 (70.7–74.9)	74.3 (72.2–76.3)	75.8# (73.8–77.8)	74.8 (72.8–76.9)	74.5 (72.5–76.5)	0.702
**General Health**	63.1 (61.5–64.7)	63.9 (62.3–65.5)	64.6 (62.9–66.1)	65.3^+#^ (63.7–66.9)	65.0 (63.4–66.6)	0.002

* Adjusted for age, sex, smoking, leisure time physical activity, total energy intake, baseline BMI and history of hypertension, diabetes, dyslipidaemia and CVD.

† statistically significantly higher (p<0.05) than Q3 (Benjamini -Hochberg correction)

+ statistically significantly higher (p<0.05) than Q2 (Benjamini -Hochberg correction).

# statistically significantly higher (p<0.05) than Q1 (Benjamini -Hochberg correction).

Vitality scores of participants belonging to the two upper quintiles of adherence to the MDP were significantly higher than those found among subjects in the two lower quintiles of adherence to this pattern) after applying the Benjamini-Hochberg correction. For mental health and physical functioning domains, also subjects in the upper levels of adherence differed significantly from subjects in the lowest level of adherence to the MDP.

**Figure 2:**
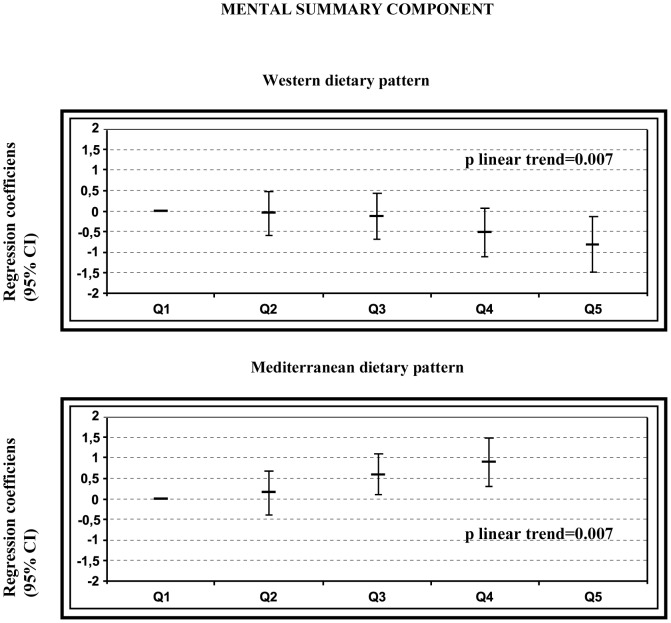
Regression coefficients of the SF-36 mental summary component according to quintiles of WDP and MDP. Adjusted for age, sex, smoking, leisure time physical activity, total energy intake, baseline BMI, and medical history of hypertension, diabetes, dyslipidaemia and CVD.

**Figure 3:**
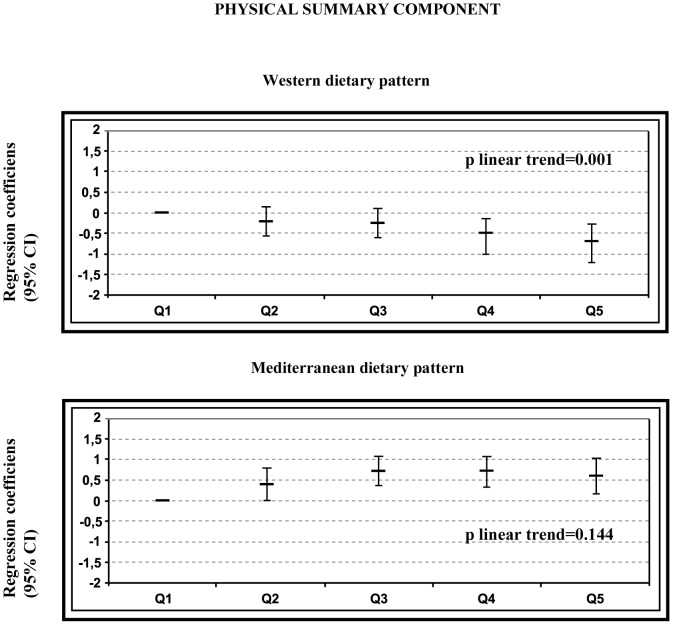
Regression coefficients of the SF-36 physical summary component according to quintiles of WDP and MDP. Adjusted for age, sex, smoking, leisure time physical activity, total energy intake, baseline BMI, and medical history of hypertension, diabetes, dyslipidaemia and CVD.

Figure 2 and Figure 3 show the regression coefficients (95% CI) for the fur upper quintiles of adherence to the dietary patterns and quality of life using the lowest quintile of the respective pattern as the reference category. The multivariate-adjusted models revealed a significant inverse association between adherence to the WDP and the two standardized summary measures of the SF-36, with a significant dose-response trend for both measures (p for trend<0.01). On the other hand, a significant direct association between adherence to the MDP and the mental summary component was found, with a significant dose-response trend (p for trend<0.01). No statistically significant association was found for the adherence to the MDP and the physical summary component.

Table 6 shows the regression coefficients (95% CI) for several sensitivity analyses that were carried out to assess possible sources of bias in the estimation of the association between baseline adherence to the WDP or the MDP and the SF-36 mental and physical components. We repeated the analyses adopting different allowed limits for total energy intake and the magnitude of the difference (quintile 5th vs. quintile 1st) for both dietary patterns and the mental and physical component remained very similar. No change in the magnitude of the association was either found after adjusting for years of education, marital status, unemployment status or alcohol intake. After the exclusion of participants with CVD, diabetes and cancer at baseline, the inverse association between extreme quintiles of WDP and the mental component was only slightly lower, although it became non-significant. We found an attenuation of the associations between baseline adherence to these dietary patterns and the mental component of the SF-36 when we repeated the analyses including 25 or 30 predefined food groups in the factor analysis.

**Table 6:** Sensitivity analyses.

Sensitivity analysis	Western pattern score			
	Mental components (Q5 *vs*. Q1)	p^*^	Physical components (Q5 *vs*. Q1)	p^*^
Energy limits: percentiles 5 to 95 (n = 10.017)	−0.7 (−0.02 to 1.4)	0.03	−0.9 (−1.4 to −0.4)	<0.001
Energy limits: percentiles 1 to 99 (n = 10.907)	−0.8 (−1.4 to −0.1)	0.01	−0.8 (−1.3 to −0.3)	0.001
Excluding participants with diabetes, CVD and cancer at baseline. (n = 10,132)	−0.5 (−1.2 to 0.2)	0.07	−0.7 (−1.2 to −0.2)	0.004
Additionally adjusted for years of education^#^	−0.8 (−1.5 to −0.2)	0.01	−0.8 (−1.3 to −0.4)	0.001
Additionally adjusted for marital status	−0.8 (−1.5 to −0.1)	0.01	−0.7 (−1.2 to −0.3)	0.001
Additionally adjusted for unemployment status	−0.8 (−1.5 to −0.1)	0.01	−0.8 (−1.3 to −0.3)	0.001
Aditionally adjusted for alcohol intake	−0.8 (−1.5 to −0.2)	0.007	−0.7 (−1.2 to −0.2)	0.002
Including 25 predefined food groups.	−0.2 (−0.9 to 0.4)	0.23	−0.9 (−1.4 to −0.4)	0.003
Including 30 predefined food groups.	−0.2 (−0.9 to 0.4)	0.23	−0.9 (−1.4 to −0.4)	0.003
	Mediterranean pattern score			
	Mental components (Q5 *vs*. Q1)	p^*^	Physical components (Q5 *vs*. Q1)	p^*^
Energy limits: percentiles 5 to 95 (n = 10.017)	0.8 (0.2 to 1.4)	0	0.7 (0.2 to 1.1)	0.003
Energy limits: percentiles 1 to 99 (n = 10.907)	0.8 (0.2 to 1.4)	0	0.6 (0.2 to 1.1)	0.03
Excluding participants with diabetes, CVD and cancer at baseline. (n = 10,132)	0.7 (0.03 to 1.3)	0.01	0.5 (0.03 to 0.9)	0.03
Additionally adjusted for years of education^#^	0.9 (0.3 to 1.5)	0.01	0.7 (0.2 to 1.1)	0.08
Additionally adjusted for marital status	0.9 (0.3 to 1.5)	0.01	0.6 (0.1 to 1.0)	0.08
Additionally adjusted for unemployment status	0.9 (0.3 to 1.5)	0.01	0.6 (0.3 to 1.0)	0.08
Aditionally adjusted for alcohol intake	0.9 (0.3 to 1.5)	0.009	0.7 (0.2 to 1.1)	0.05
Including 25 predefined food groups.	0.7 (0.1 to 0.3)	0.01	0.6 (0.2 to 0.1)	0.002
Including 30 predefined food groups.	0.7 (0.1 to 1.30)	0.01	0.6 (0.2 to 1.1)	0.002

p^*^: linear trend; ^#^: Years of education: 3, 4, 5, 6 and 9 years of University education.

Regression coefficients (95% confidence intervals)^ *^ of the SF-36 mental and physical summary components according to quintiles of Western pattern and Mediterranean pattern score.

## Discussion

In a large Mediterranean cohort, two main dietary patterns were identified: the Western Dietary Pattern (WDP) and the Mediterranean Dietary Pattern (MDP). Adherence to the WDP was associated with poorer scores in the 2 standardized summary measures of quality of life (the mental and the physical summary measures) and in all the 8 domains of the SF-36. On the contrary, adherence to the MDP was associated with higher quality of life scores in the mental summary component and in 4 of the 8 individual domains of the SF-36 (vitality, mental health, physical functioning and general health).

Dietary patterns derived with an a posteriori approach have been investigated in relation to many health outcomes [35], [36], but to our knowledge this is the first time that they have been related to quality of life. Although HRQL is a perceived health measure rather than a biological measure, self-related health status has been shown to be a powerful predictor of mortality in the long term [37].

Our results suggest that a dietary pattern characterized by frequent consumption of fast-food, red and processed meats, high-fat dairy, processed foods, eggs and commercial bakery seems to be deleterious for quality of life, whereas a dietary pattern rich in vegetables, fish, fruits, poultry and olive oil is directly associated with a better quality of life. Moreover, an adjusted difference of −3.5 points in the mean values of vitality was obtained when participants with the highest adherence to the WDP were compared to those with the lowest adherence. Similar absolute difference was observed as we compared participants in the extreme quintiles of adherence to the MDP.

There is a debate on how to define clinically meaningful differences on the SF-36 scores. Changes in 3-, 5-, and 10- points have been suggested as being clinically meaningful for clinical populations [38]. Although few studies have examined this issue directly, several investigators have raised the question of whether individuals with more severe impairments in HRQL at baseline might require a greater absolute change in their quality of life in order to consider this improvement clinically meaningful than those with less severe impairments at baseline [34].

Our cohort included healthy and relatively young adults with good average quality of life. In this context it is difficult to find large differences because most participants perceive themselves in good health. But even in this setting we did find significant associations between dietary patterns and quality of life. It seems logical to think that in another context with worse quality of life (e.g. patients with a chronic disease) differences in quality of life related to dietary patterns could be expected to be even higher.

Scientific literature has consistently shown the deleterious effect of the WDP, a food pattern described in several large American cohort studies. Epidemiological studies have reported a detrimental effect on weight gain, obesity and insulin resistance [39], [40]. Moreover, this pattern has also been associated with the risk of CVD, endothelial dysfunction and higher level of pro-inflammatory cytokines [26], [41], [42]. The high content of saturated, trans-unsaturated fatty acids, and refined sugars usually present in the foods that characterize the Western diet are candidates to be the responsible agents for the reported associations [43]. The adverse effects of saturated, trans-unsaturated fats on CVD are thought to be mediated by increases in plasma concentrations of LDL-cholesterol, reductions in HDL-cholesterol, pro-inflammatory changes, endothelial dysfunction, and possibly by insulin resistance and displacement of essential fatty acids from membranes [44], [45]. On the other hand, few epidemiological studies have addressed the long-term health impact of dietary patterns characterized also with high-sugar foods like the WDP, but diets with low glycemic load have been associated with a decreased risk of coronary heart disease, [46] lower level of pro-inflammatory cytokines and a better lipid profile [47].

With regard to mental quality of life, several studies have associated the adherence to a Western-style dietary pattern high in fats and refined sugars to a higher risk of depression and anxiety or to the presence of mental symptoms [48], [49]. A possible explanation for this finding is that the Western dietary pattern could decrease the levels of the brain-derived neurotrophic factor (BDNF) within a short period of time as some animal studies have shown [50]. In fact, the Western dietary pattern is able to increase the production of pro-inflammatory cytokines that inhibit the production of this neurothophin. BDNF is a crucial mediator of neuronal vitality and function [51]. Therefore, it is likely that a dietary pattern rich in commercial bakery, sugary beverages and fast-food could increase the vulnerability to some mental or neurological disorders or act negatively on mental quality of life. All of these physiological processes may also be influencing physical and mental quality of life in healthy populations.

Contrarily to the WDP, the adherence to a traditional MDP has been inversely associated with the incidence of CVD, obesity or the metabolic syndrome [52], [53]. The Mediterranean Diet is rich in nutrients such as vitamins, minerals, antioxidants, fibre, omega-3 fatty acids (from fish) and monounsaturated fatty acids (from olive oil) whose beneficial effects on health have been widely demonstrated [54]. Several biological and physiological mechanisms could explain the beneficial effect of the MDP on physical health. A greater adherence to a MDP has been inversely associated with a reduction in low-grade inflammatory status, better endothelial function and lower insulin resistance [41], [55], [56]. All these factors are thought to lead not only to a lower risk of chronic diseases, but also to a better metabolic control of already established diseases. Furthermore, this dietary pattern has also been related with a better mental health status. Several studies have reported a lower risk of neurodegenerative diseases, depression or mental disorders among subjects with a better adherence to the traditional MDP [10], [57]. Some biological mechanisms to explain this association are based on the role that long chain omega-3 fatty acids are thought to play in the central nervous system, including the dynamic structure and fluidity of neural membrane and beneficial influences on serotonin transport [58]. On the other hand B vitamins and folate are involved in 1-carbon metabolism that acts in several methylation reactions, such as those related to the synthesis of serotonin and other monoamine neurotransmitters [59].

Several limitations in our study need to be addressed. Although the validity and reliability of the food-frequency questionnaire have been extensively evaluated, as well as the validity of the SF-36 questionnaire, some degree of misclassification may exist in the dietary and in the outcome assessment and over- or under-estimation of true intake and health status could have biased our estimates. However, being the miss-classification most probably non-differential, the bias would be probably towards the null. Therefore, we do not believe that misclassification might be an alternative explanation of the significant associations we identified. On the other hand, diet was ascertained at baseline and quality of life after 4-years of follow-up. Therefore we acknowledge that baseline scores of quality of life were unknown. It could be possible to speculate that a high quality of life at baseline might lead to a better general situation with healthier life-style including the adoption of healthier food habits. Thus, the effect exerted for both dietary patterns on HRQL could be in part due to better/worse baseline physical and mental health status. In any case, we have adjusted for a wide array of baseline characteristics that could be considered markers of quality of life such as smoking, physical activity, energy intake, body size or medical history of chronic diseases [60]–[62]. Thus, we have probably reduced the baseline heterogeneity among participants.

Dietary patterns were constructed only at baseline, so changes in dietary patterns during the follow-up period were not considered. Further studies are needed to complete this assessment using repeated measurements of diet.

Also, it is generally accepted that socio-economic status influences dietary habits as well as human health. However, the participants in our cohort were restricted to university graduates (responsible, highly motivated, and many of them former students of a private university). So, we consider that the sample is fairly homogeneous regarding socio-economic status (medium-high). Moreover, restriction is an excellent technique for preventing or at least reducing confounding by known factors, and it is recommended by methodologists because restriction is usually more effective than multivariable adjustment to control for potential confounding [63]. Finally, our results did not change after the adjustment for several socioeconomic factors such as years of education, employment status or marital status in our sensitivity analyses.

Another fact to take into account is that quality of life is a complex concept with various dimensions. Nevertheless, the use of the SF-36 questionnaire for evaluating the physical and mental dimensions of quality of life is generally accepted, and its validity and reliability have been demonstrated in many population-based studies [64], including the validated version translated into Spanish [65].

Some strengths of our study also deserve to be mentioned. They include its large sample size, its long-term follow-up, the multiple adjustments of our estimates for a variety of major potential confounders and the existence of published validation studies of our assessments.

## Conclusions

Self-perceived mental and physical quality of life was inversely associated with the adherence to a WDP, and directly associated with the adherence to a MDP. Nevertheless, replication of these findings in longitudinal studies, with the inclusion of a baseline determination of the initial quality of life is required in order to confirm these associations.
